# Narrative exposure therapy for posttraumatic stress disorder associated with repeated interpersonal trauma in patients with severe mental illness: a mixed methods design

**DOI:** 10.3402/ejpt.v7.32473

**Published:** 2016-09-21

**Authors:** Maria W. Mauritz, Betsie G. I. Van Gaal, Ruud A. Jongedijk, Lisette Schoonhoven, Maria W. G. Nijhuis-van der Sanden, Peter J. J. Goossens

**Affiliations:** 1 GGNet Mental Health Care Center, Warnsveld, The Netherlands; 2 Radboud university medical center, Radboud Institute for Health Sciences, IQ healthcare, Nijmegen, The Netherlands; 3 Foundation Centrum '45, Oegstgeest, The Netherlands; 4 Arq Psychotrauma Expert Group, Diemen, The Netherlands; 5 Faculty of Health Sciences, University of Southampton, Southampton, United Kingdom; 6 Dimence Group, Center for Mental Health Care, SCBS Bipolar Disorders, Deventer, The Netherlands; 7 University Centre for Nursing and Midwifery, Department of Public Health, Faculty of Medicine and Health Sciences, Ghent University Ghent, Belgium; 8 GGZ-VS, Institute for Education of Clinical Nurse Specialist in Mental Health, Utrecht, The Netherlands

**Keywords:** Physical abuse, sexual abuse, posttraumatic stress disorder, schizophrenia, mood disorder, personality disorder, flexible assertive community treatment, mixed methods, repeated measures, in-depth interview

## Abstract

**Background:**

In the Netherlands, most patients with severe mental illness (SMI) receive flexible assertive community treatment (FACT) provided by multidisciplinary community mental health teams. SMI patients with comorbid posttraumatic stress disorder (PTSD) are sometimes offered evidence-based trauma-focused treatment like eye movement desensitization reprocessing or prolonged exposure. There is a large amount of evidence for the effectiveness of narrative exposure therapy (NET) within various vulnerable patient groups with repeated interpersonal trauma. Some FACT-teams provide NET for patients with comorbid PTSD, which is promising, but has not been specifically studied in SMI patients.

**Objectives:**

The primary aim is to evaluate NET in SMI patients with comorbid PTSD associated with repeated interpersonal trauma to get insight into whether (1) PTSD and dissociative symptoms changes and (2) changes occur in the present SMI symptoms, care needs, quality of life, global functioning, and care consumption. The second aim is to gain insight into patients’ experiences with NET and to identify influencing factors on treatment results.

**Methods:**

This study will have a mixed methods convergent design consisting of quantitative repeated measures and qualitative semi-structured in-depth interviews based on Grounded Theory. The study population will include adult SMI outpatients (*n*=25) with comorbid PTSD and receiving NET. The *quantitative* study parameters will be existence and severity of PTSD, dissociative, and SMI symptoms; care needs; quality of life; global functioning; and care consumption. In a longitudinal analysis, outcomes will be analyzed using mixed models to estimate the difference in means between baseline and repeated measurements. The *qualitative* study parameters will be experiences with NET and perceived factors for success or failure. *Integration* of quantitative and qualitative results will be focused on interpreting how qualitative results enhance the understanding of quantitative outcomes.

**Discussion:**

The results of this study will provide more insight into influencing factors for clinical changes in this population.

**Highlights of the article:**

- Nearly 50% of severely mentally ill (SMI) patients have been exposed to repeated violence and 30% is suffering from PTSD.
- Narrative exposure therapy (NET) is designed for PTSD related to repeated trauma and proved effective in refugees and other vulnerable patient groups.
- NET is provided to SMI patients with PTSD and the first clinical outcomes are promising.
- This study will analyze symptom changes after NET in relation to patients’ experiences with this treatment.

Despite the large attention in research for trauma and posttraumatic stress disorder (PTSD), the interest in the prevalence and treatment options for trauma and PTSD in severely mentally ill (SMI) populations is scarce, albeit growing. Research results show that documentation of trauma and trauma symptoms is exceptionally low in medical records of SMI patients. Improved recognition is needed to provide adequate treatment and meaningful services to this vulnerable population (Cusack, Grubaugh, Knapp, & Frueh, 2006; De Bont et al., 2013; Kilcommons & Morrision, 2005; Mueser et al., 1998, 2004). In a recently conducted review on trauma and PTSD in SMI, the population weighted mean prevalence rates are, respectively, physical abuse 47%, sexual abuse 37%, and PTSD 30%. (Mauritz, Goossens, Draijer, & Van Achterberg, 2013). These prevalence rates are significantly higher than in the general population where the prevalence of physical abuse is 21% and sexual abuse is 23% (Briere & Elliott, 2003), and the lifetime prevalence of PTSD is estimated to be 6.8% among adult Americans (Kessler, Berglund, Demler, Jin, & Walters, 2005; Kessler, Chiu, Demler, Merikangas, & Walters, 2005) and 7.4% in de Netherlands (De Vries & Olff, 2009). Repeated interpersonal trauma such as physical and sexual abuse may result in PTSD with dissociative symptoms, which is classified as PTSD, dissociative subtype in DSM-5 (American Psychiatric Association [APA], 2013). Interpersonal trauma and comorbid PTSD both have a negative influence on the course of the SMI (Mauritz et al., 2013; Mueser, Goodman, Rosenberg, & Trumbetta, 2002). Trauma exposure itself is strongly related to several other psychiatric disorders. Childhood trauma, for instance, is identified as a causal factor in the development of psychotic disorders (Read, Van Os, Morrison, & Ross, 2005). It is also shown that PTSD in patients with SMI can be treated effectively using the following treatment options: cognitive restructuring (Lu et al., 2009; Mueser et al., 2008), prolonged exposure (PE) (Frueh et al., 2009; Van Minnen, Harned, Zoellner, & Mills, 2012) or eye movement desensitization reprocessing (EMDR) (Van Den Berg & Van Der Gaag, 2012). In a recently conducted randomized controlled trial, both PE and EMDR treatment appeared effective, safe, and feasible in patients with severe psychotic disorders and comorbid PTSD (Van den Berg et al., 2015).

Narrative exposure therapy (NET) is a relatively new trauma-focused treatment. It has been shown effective in vulnerable groups like refugees and other patients with a history of repeated trauma exposure including patients with borderline personality disorder (BPD) or major depression and comorbid PTSD. NET appeared to be well tolerated in these groups (Gwozdziewycz & Mehl-Madrona, 2013; Neuner et al., 2008; Pabst et al., 2014; Robjant & Fazel, 2010; Stenmark, Catani, Neuner, Elbert, & Holen, 2013). NET is indicated for PTSD as a result of repeated trauma, usually as a result of interpersonal violence.

## Objectives

The primary aim of this study is to evaluate NET in SMI patients with comorbid PTSD associated with repeated interpersonal trauma to get insight into whether (1) the PTSD and present dissociative symptoms changes and (2) changes occur in the present SMI symptoms, care needs, quality of life, global functioning, and care consumption. The second aim is to gain insight into patients’ experiences with NET and to identify influencing factors on treatment results in terms of symptom changes, care needs, and quality of life.

## Methods

This study uses a mixed methods convergent design: a quantitative repeated measures design and qualitative methods consisting of a Grounded Theory design. The aim of a mixed methods design is to integrate quantitative and qualitative components to obtain additional knowledge (Boeije, Slagt, & Van Wesel, 2013; Creswell & Zhang, 2009). In this study, integration will be focused on interpreting how qualitative outcomes regarding patients’ experiences with NET enhance the understanding of the quantitative clinical outcomes.

## Study population

The study population consists of adult (aged 21–65 years) SMI patients receiving flexible assertive community treatment (FACT). SMI is defined as the presence of schizophrenia-spectrum disorder, mood disorder, or personality disorder and decreased global functioning, which is operationalized by a Global Assessment of Functioning (GAF)-score <60 during 2 or more years (Kessler et al., 2003; Mauritz et al., 2013; Ruggeri, Leese, Thornicroft, Bisoffi & Tansella, 2000). FACT includes coordinated multidisciplinary treatment (i.e., pharmacotherapy, cognitive behavioral therapy, and other relevant evidence-based interventions) and collaborative care (i.e., case management and nursing care) for patients in a stable phase. Unstable patients receive intensive assertive outreaching care delivered by more team members according to the principle of shared caseload (Drukker, Visser, Sytema, & Van Os, 2013; Van Veldhuizen, 2007).

Patients with a history of repeated interpersonal trauma (physical and/or sexual abuse) and with comorbid PTSD are offered NET by trained FACT-team therapists. Participants will be recruited from all FACT-teams from 10 community mental health teams at a large mental health institute in the east Netherlands. Inclusion will start at the beginning of 2016 and will last to the end of 2017.

Potential participants will obtain oral and written information about the study from their treating psychiatrist and the primary investigator. They will be asked to sign an informed consent form if they are willing to participate.

### Inclusion criteria

In order to be eligible to participate in this study, a patient must meet the following criteria for the existence of SMI: (1) a diagnosis of schizophrenia (DSM-5 295.90), or schizoaffective disorder (DSM-5 295.70), bipolar disorder type I (DSM-5 296.40–46 or 296.50–56) or type II (DSM-5 296.89), or major depressive disorder (DSM-5 296.20–26 or 296.30–36) (APA, 2013) according to the Mini-International Neuropsychiatric Interview (M.I.N.I.-plus) (Sheehan et al., 1998) or personality disorder (DSM 5 301.xx) according to the Structured Clinical Interview for DSM-IV Personality Disorders SCID-II (First, Spitzer, Gibbon, & Williams, 1995) and each has a GAF-score (APA, 2000) <60 during ≥2 years according to the chart diagnosis; (2) a trauma history including repeated physical and/or sexual abuse according to the Life Events Checklist for DSM-5 (LEC-5) (Boeschoten, Bakker, Jongedijk, & Olff, 2014a); and (3) PTSD according to the Clinician-Administered PTSD Scale for DSM-5 (CAPS-5) (Boeschoten et al., 2014).

### Exclusion criteria

A potential patient who meets any of the following criteria will be excluded from participation in this study: (1) the provision of other trauma-focused treatment in the past year, (2) the existence of an antisocial personality disorder, (3) the existence of a dissociative identity disorder, and (4) the provision of involuntary treatment following the Dutch Mental Health Law.

### Withdrawal of participants

Participants can leave the study at any time for any reason if they wish to do so without any consequences. In case participants do leave the study, a maximum of five leaving participants will be replaced before the end of 2017. Patients who do not complete their NET will stay in the study, unless they want to leave the study. Withdrawal will be monitored and analyzed.

### Sample size

We expect to be able to include 25 participants within 2 years. This number of participants will be sufficient to quantify the clinical changes as assessed by the diagnostic instruments used in this population. For qualitative in-depth analysis, the number of 25 participants will be sufficient and enables integral analysis by comparing quantitative and qualitative results within each individual and the whole group.

## Treatment with narrative exposure therapy

Schauer, Neuner, and Elbert (2011) describe the proved effective main elements of NET as follows: (1) an active chronological reconstruction of the autobiographical memory, (2) PE to the traumatic memory with full activation of the emotional fear network through detailed narration and imagination of the traumatic event, (3) meaningful linkage and integration of physiological, sensory, cognitive, and emotional responses to the persons time, space, and life context, (4) cognitive re-evaluation of behavior and patterns, reinterpretation of the meaning content through reprocessing of fearful and traumatic events, with completion and closure, (5) revisiting of positive life experiences, and (6) regaining of dignity through satisfaction of the need for acknowledgment through “testifying” (Jongedijk, 2014; Schauer et al., 2011).

The provision of NET is outlined as follows: Session 1: psycho-education; Session 2: the lifeline which is symbolized by a rope with a coiled end, stones (traumatic events) and flowers (positive life experiences) in time sequences; Session 3: start of the narration beginning at birth and continuing through to the first traumatic event; Session 4 and subsequent sessions: rereading of the narrative collected in previous sessions and continuing the narration of subsequent life and traumatic events; and final session: rereading and signing the whole document. The average number of the weekly 90-min sessions varies from 6 to 16 (Jongedijk, 2014; Jongedijk & Mauritz, 2016; Schauer et al., 2011). NET is provided by both nurse practitioners and psychologists who are all thoroughly trained by official qualified and certified trainers and who receive video supervision by a certified psychologist or nurse practitioner. The weekly NET sessions will be arranged and conducted at the regional mental health care center location.

## Data collection

### Quantitative measurements

Patients will participate in the study for 10 months. At pre-treatment (T0), a trained and supervised research assistant will collect demographic information from the electronic record and assess baseline measurements of all variables. These variables include trauma history, PTSD diagnosis and symptoms, dissociative symptoms, SMI diagnosis and symptoms, care needs, quality of life and global functioning. At post-treatment 1 month after NET (T1) and after 7 months follow-up (T2), clinical outcomes including PTSD symptoms, dissociative symptoms, SMI symptoms, care needs, quality of life, and global functioning are assessed again (see Table 1).

**Table 1:** Measurements in scheme

	T0 inclusion and before intervention	T1 1 month after finalizing intervention	T2 7 months follow-up
Demographics	Collected via the electronic record		
Care consumption		Collected via the electronic record	
Trauma history	LEC-5		
PTSD symptoms	CAPS-5	CAPS-5	CAPS-5
Dissociative symptoms	DES	DES	DES
SMI diagnosis	M.I.N.I.-plus or SCID-II		
SMI symptoms	HoNOS	HoNOS	HoNOS
Care needs	CAN	CAN	CAN
Quality of life	MANSA	MANSA	MANSA
Global functioning	GAF	GAF	GAF

Care consumption and prescribed psychiatric medications will be collected via the electronic record. These data will be analyzed for the following period: 3 months before T0 up to and including T2.

#### Demographic information

Demographic variables include gender, age, marital status, level of completed education, number and duration of traumatic event types (emotional, physical, and/or sexual abuse in childhood and/or adulthood), primary SMI diagnosis, duration of illness, prescribed medication, and experiences with forced hospitalization and seclusion. These variables are largely collected via the electronic record and based on the inclusion interview outcomes.

#### Care consumption

Care consumption is defined as the number and time duration in minutes of contacts with FACT-team members and prescribed psychiatric medications (type and doses).

### Instruments

#### Life Events Checklist for DSM-5

The LEC is developed concurrently with the CAPS to facilitate the diagnosis of PTSD and is a measure of exposure to 17 potentially traumatic events. The LEC is evaluated in college undergraduates and combat veterans by Gray, Litz, Hsu, and Lombardo (2004). In this study, the LEC had temporal stability and convergent validity and was strongly associated with PTSD symptoms in a clinical sample. The LEC-5 connects to the CAPS-5 and has some minimal changes compared with the original LEC (Weathers et al., 2013b). The LEC-5 will be administered after positive screening for repeated trauma exposure on the Trauma Screening Questionnaire (TSQ) (Brewin et al., 2002; De Bont et al., 2015) and PTSD on the PTSD checklist for the DSM-5 (PCL-5) (Boeschoten, Jongedijk, & Olff, 2014b; Weathers et al., 2013). The Dutch version of the LEC-5 will be used in this study (Boeschoten et al., 2014a).

#### Dissociative Experiences Scale

Dissociative symptoms are measured using the Dissociative Experiences Scale (DES), which consists of 28 items and records the severity of dissociative symptoms. The total score for the DES can range from 0 to 100. A total score over 40 suggests a dissociative disorder (Bernstein & Putnam, 1986; Carlson et al., 1993). The Dutch version of the DES discriminates dissociative disorders from other psychiatric disorders and appeared as a reliable and valid instrument (Draijer & Boon, 1993; Ensink & Van Otterloo, 1989).

#### Clinician-Administered PTSD Scale for DSM-5

The CAPS is a structured diagnostic interview designed for PTSD according to DSM-IV (Blake et al., 2005). The CAPS is considered as the gold standard clinical interview to establish the diagnosis of PTSD (Weathers, Keane & Davidson, 2001). The CAPS-5 is based on the original CAPS for DSM-IV but has several important revisions based on the corresponding DSM-5 criteria for PTSD. This 30-item structured interview can be used to make current (past month) and lifetime diagnosis of PTSD and assess PTSD symptoms over the past week (Weathers et al., 2013a). The Dutch version of the CAPS-5 will be used (Boeschoten et al., 2014).

#### Mini-International Neuropsychiatric Interview

The M.I.N.I.-plus is a short structured diagnostic interview, developed jointly by psychiatrists and clinicians in the United States and Europe, for DSM-IV and ICD-10 psychiatric disorders. The M.I.N.I.-plus was designed as a brief structured interview for the major Axis I psychiatric disorders in DSM-IV and ICD-10. Validation and reliability studies show that the M.I.N.I.-plus has acceptably high validation and reliability scores and can be administered in a short period of time (mean 18.7±11.6 min, median 15 min). It can be used by clinicians after a brief training session (Sheehan et al., 1998). The Dutch version of the M.I.N.I.-plus is tested in a selected group of Dutch psychiatric patients. Based on initial experiences and results, Van Vliet and De Beurs (2007) conclude that the good psychometric characteristics of the M.I.N.I.-plus make it a good choice for research purposes. Because of its brevity (20–30 min), the interview seems to be especially convenient for diagnosing psychiatric patients in everyday clinical practice (Van Vliet & De Beurs, 2007).

#### Structured Clinical Interview for DSM-IV Personality Disorders

The SCID-II is a clinician-administered semi-structured interview for diagnosing the Axis II personality disorders of the DSM-III-R. It was designed to provide a rapid clinical assessment of personality disorders without the loss of reliability or validity (First et al., 1995). After the publication of the DSM-IV in 1994, the SCID-II has been revised to meet the DSM-IV criteria (SCID-II version 2.0). Maffei et al. (1997) assessed the interrater reliability and internal consistency of the SCID-II 2.0 and concluded that this version of the SCID-II has adequate interrater and internal consistency reliability. In the Netherlands, the reliability of the Dutch version of the SCID-II was further investigated with regard to the test–retest interrater reliability. Test–retest interrater reliability for the presence or absence of any personality disorder was fair to good and was higher than values found in previous short-interval test–retest studies with the SCID-II for DSM-III-R (Weertman, Arntz, Dreessen, Velzen, & Vertommen, 2003).

#### Health of the Nation Outcome Scale

The Health of the Nation Outcome Scale (HoNOS) has been developed for assessing the effectiveness of mental health services for patients with SMI. It covers a wide range of areas and has good criterion and concurrent validity (Orrell, Yard, Handysides, & Schapira, 1999). Rees, Richards, and Shapiro (2004) assessed the usefulness of the HoNOS in measuring change in a community mental health population and concluded that a change of 3–4 points was statistically significant and useful for tracking clinical improvement. The Dutch translation of the HoNOS has been studied and provided insight at both individual and group level into the seriousness of problems and into occurred changes. It has reasonably good psychometric qualities, can be administrated in a short time, is not dependent on psychiatric diagnosis or language, and is used by both clinicians and patients (Mulder et al., 2004). The HoNOS is designed for the heterogeneous SMI population and is suitable to evaluate possible symptom changes within the individual and also at group level.

#### Camberwell Assessment of Needs

The Camberwell Assessment of Need (CAN) is commonly used for comprehensive needs assessment in mental health services. It is a valid, reliable, and usable measuring instrument to assess the care need of SMI patients (Phelan et al., 1995). Wennström, Sorbom, and Wiesel (2004) concluded after exploratory factor analysis that the CAN represents three homogeneous dimensions: functional disability, social loneliness, and emotional loneliness. The summary scores of items corresponding to functional disability and social health might be more reliable and more sensitive to changes over time than the standard CAN summary scores.

#### Manchester Short Assessment of quality of life

The MANSA is a short instrument for assessing quality of life in people with mental illness. This instrument measures satisfaction of life as a whole and with life domains (Priebe, Huxley, Knight, & Evans, 1999). The MANSA was examined with regard to reliability and construct validity in patients with SMI. Internal consistency was adequate (α=0.81). The construct validity of the scale was satisfactory in this study (Björkman & Svensson, 2005).

#### Global Assessment of Functioning Scale

The GAF Scale measures psychological, social, and occupational functioning. This scale considers a hypothetical continuum of mental health illness and does not include impairment in functioning as a result of physical or environmental limitations. It is used for the DSM-IV classification (APA, 2000).

### Qualitative semi-structured interviews

To investigate participants’ experiences with NET and to identify possible influencing factors for success or failure of NET, participants will be interviewed. Sensitizing concepts are (1) experiences during NET, (2) symptom changes, (3) care needs, (4) quality of life and daily life functioning, and (5) influencing factors and meaning. Based on these concepts, the following topics are derived and listed: ad 1: therapeutic relationship, lifeline, narration, exposure, and the treatment effects in daily life; ad 2: experienced symptoms; ad 3: perceived care needs; ad 4: perceived quality of life and effects on daily functioning; and ad 5: experienced success or failure and significance for meaningfulness in daily life (see Supplementary file).

The interviews will be held 3 months after finalizing NET (see Table 2). The maximum length of the interviews will be 60 min. One independent researcher (first author) will interview all participants. Interviews will be audiotaped and typed out verbatim. The interviewer will be blinded for measurement results to provide a neutral attitude and prevent bias based on foreknowledge. Interviews will be held on the known FACT location or at the patient's home. Participants can stop the interview at any moment. The audio files and written content of the interview results are confidential.

**Table 2:** The mixed methods convergent design in scheme.

	0	1	2	3	4	5	6	7	8	9	10
	
Months		NET			Follow-up						
Data collection	T0				T1			I			T2
Quantitative: measurements	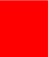				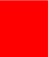						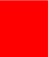
Qualitative: semi-structured interviews								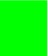			

## Analysis

### Quantitative analysis

Descriptive analysis will be used to describe the patient characteristics and the repeated (outcome) measures. To analyze the clinical outcomes of the NET, mixed models will be used in a longitudinal analysis to estimate the effect within the individual and the whole group, taking into account the repeated measures.

### Qualitative analysis

The qualitative data analysis forms a cyclic process, with the interim analyzes modifying subsequent interviews. This procedure is well known as the constant comparative method. The texts of the interviews will be transcribed literally and then entered in the ATLAS.ti computer program for qualitative text analysis (Muhr & Friese, 2004). Then, they will be started with open coding: small text fragments will be labeled. This label or code has a descriptive nature and closely follows the text. At the same time, questions and remarks about ambiguities in the text or possible interpretations will be described in brief notes. The next analysis stage will exist of joining the small text fragments without losing the original codes and labeling these fragments with a higher level of abstraction. These codes will be not only descriptive but also interpretative. During this process, the similarities and differences between interviews will be explored according to the principles of the constant comparative analysis. During the third analysis stage, text fragments will be further joined, categorized, and described. These categories will reflect the dominant themes and possible relevant determinants. The interviews will be independently coded by two researchers.

#### Methodological objectivity

The methodological objectivity will be enhanced by (1) purposive sampling confined to SMI patients who have recently received NET treatment for comorbid PTSD, (2) a neutral attitude in asking questions and in orally checking the findings with the respondent, (3) researcher triangulation: minimization of researcher bias by independent coding and analyzing interviews by two researchers, (4) member check in order to assess the accuracy of the representation of the participant's subjectivity, (5) peer debriefing with the whole research group during data collection and analysis, (6) peer debriefing with two independent NET therapists and two FACT-team members who care for the participants, (7) transparency and reproducibility through careful documentation of all steps of the research process. Interviews are tape recorded and verbatim transcribed. The raw material is saved and stored in its entirety to be available for verification purposes.

### Integrated analysis

Integration of the quantitative and qualitative results will be focused on interpreting how the qualitative results enhance the understanding of the quantitative outcomes. The quantitative and qualitative results of the experienced symptoms (i.e., PTSD, dissociation, and present primary disorder), perceived care needs, and quality of life will be compared within each individual. Additional, this will be analyzed at group level. Finally, congruent and discrepant findings will be interpreted.

### Ethics

The study will be conducted with ethical principles that are consistent with the Declaration of Helsinki, amended by the 59th WMA General Assembly, Seoul, October 2008. The ethical approval for conducting the “NET for PTSD in SMI patients” study was provided by the Committee on Research Involving Human Subjects, Arnhem-Nijmegen (number 1843-2015).

## Discussion

NET has been shown to be effective in vulnerable groups, and first clinical results in SMI patients with comorbid PTSD receiving FACT have been promising. To evaluate and improve clinical practice, only relying on quantitative measures might not suffice as it lacks sensitivity for this specific population. Moreover, the mixed methods approach further enriches the quantitative data with qualitative interviews addressing patients’ perspectives on effectiveness, barriers, and facilitating factors.

## Registration

This study protocol is registered in The Netherlands National Trial Register (NTR) with number NTR5714.
